# Problematic Social Media Use and Its Associations with Loneliness, Bullying Perpetration, and Negative Body Image Among Adolescents: The Mediating Roles of Secure Attachment and Resilience

**DOI:** 10.3390/bs16091470

**Published:** 2026-08-24

**Authors:** Mehmet Akif Kay, İlhan Çiçek, Fırat Ünsal, Mehmet Sıddık Vangölü, Zafer Korkmaz, Samet Makas, Juan Gómez-Salgado, Murat Yıldırım

**Affiliations:** 1 Department of Child Care and Youth Services, Vocational School of Social Sciences, Batman University, Batman 72100, Türkiye; 2 Health College, Batman University, Batman 72100, Türkiye; 3 Department of Psychology, Faculty of Science and Arts, Bitlis Eren University, Bitlis 13000, Türkiye; 4 Faculty of Education, Van Yüzüncü Yıl University, Van 65080, Türkiye; 5 Faculty of Education, Guidance and Psychological Counselling, Sakarya University, Sakarya 54050, Türkiye; 6 Department of Medicine and Public Health, Faculty of Labour Sciences, University of Huelva, 21071 Huelva, Spain; 7 Safety and Health Postgraduate Program, Universidad Espíritu Santo, Guayaquil 092301, Ecuador; 8 Department of Psychology, Faculty of Science and Letters, Agri Ibrahim Cecen University, Ağrı 04100, Türkiye; 9 Department of Psychology, Faculty of Arts and Sciences, Haliç University, İstanbul 34060, Türkiye; 10 Psychology Research Centre, Khazar University, Baku AZ1096, Azerbaijan

**Keywords:** problematic social media use (PSMU), loneliness, bullying perpetration, negative body image, secure attachment, resilience, adolescents

## Abstract

**Background**: Problematic social media use (PSMU) has become increasingly prevalent among adolescents and is consistently associated with a wide range of adverse mental health outcomes, underscoring its growing significance as a contemporary psychosocial and developmental concern. However, empirical research examining protective psychological factors associated with relationships between PSMU and psychological difficulties remains limited. The present study examined whether resilience and secure attachment were associated with the relationships between PSMU and loneliness, bullying perpetration, and negative body image among adolescents. **Methods**: Participants were 712 adolescents aged between 13 and 18 years (M_age_ = 15.33, SD = 1.13; 61.1% females, 38.9% males). Standardized scales were used to assess problematic social media use, secure attachment, resilience, bullying perpetration, loneliness, and negative body image. **Results**: The findings indicated that higher levels of problematic social media use (PSMU) were significantly associated with lower levels of secure attachment and resilience, alongside higher levels of loneliness, bullying perpetration, and negative body image. Secure attachment and resilience were also significantly associated with lower loneliness, bullying perpetration, and negative body image. Moreover, significant indirect associations were observed between PSMU and loneliness, bullying perpetration, and negative body image through secure attachment and resilience. **Conclusions**: These findings suggest that secure attachment and resilience may represent important psychological resources associated with the relationships between PSMU and psychosocial difficulties during adolescence. Given the cross-sectional design of the study, these findings should be interpreted as statistical associations rather than causal processes or mechanisms.

## 1. Introduction

Adolescence constitutes a pivotal developmental period involving substantial changes across physical, cognitive, and socio-emotional domains, alongside intensified processes of identity formation, all of which may carry substantial implications for psychological well-being and social adjustment. In contemporary societies, rapid technological advancement has led to the pervasive use of online communication tools, profoundly influencing adolescents’ social interactions and identity development (Belfort et al., 2026; Boursier et al., 2020; Li et al., 2024; Lu et al., 2025; Sala et al., 2024). A growing body of literature suggests that problematic social media use—characterized by excessive, poorly controlled, and maladaptive engagement—is associated with multiple adverse outcomes in adolescents’ mental health and social functioning.

PSMU has been consistently associated with elevated levels of depressive symptoms, psychological stress, and anxiety (Diaz et al., 2025; Eddy Ives et al., 2025), heightened loneliness (Ünsal et al., 2025; Yam et al., 2024), social appearance anxiety (Boursier et al., 2020), body image disturbances (Çimke & Yıldırım Gürkan, 2023), and sleep problems (Korkmaz et al., 2023). Collectively, these findings suggest that problematic social media engagement is associated with a broad range of adverse psychosocial outcomes and may negatively influence adolescents’ self-perceptions and social adjustment.

These difficulties also extend into adolescents’ peer relations, where PSMU has been associated with greater engagement in bullying perpetration (Yüksel Doğan & Demircioğlu, 2025). Conversely, other lines of evidence suggest that being exposed to bullying may itself contribute to problematic internet use, potentially through self-regulatory difficulties (Cortazar et al., 2025) or heightened loneliness (Eid et al., 2023). Together, these findings point to a complex, possibly bidirectional relationship between bullying and PSMU, although perpetration and victimization represent distinct constructs that should not be conflated. The present study focuses specifically on adolescents’ engagement in bullying perpetration.

Despite the expanding literature, studies that simultaneously examine PSMU, bullying perpetration, loneliness, and body image within an integrated framework remain scarce. Conceptually, PSMU is characterised by loss of control over use, preoccupation with social media, and persistence in use despite adverse consequences (Bányai et al., 2017). Empirical evidence suggests that adolescents with elevated loneliness may resort to social media to compensate for deficits in offline social connections (Wu et al., 2024). However, such compensatory use often fails to meet underlying social needs and may intensify loneliness by reducing face-to-face interactions and amplifying biased social comparison processes (Wu et al., 2024; Yam et al., 2024).

The association between PSMU and negative body image represents another important concern during adolescence. Highly visual social media platforms, such as Instagram, expose adolescents to idealized and heavily edited representations of appearance and beauty standards, thereby fostering frequent upward social comparisons that may contribute to body dissatisfaction and negative affect (Chua & Chang, 2016; Saiphoo & Vahedi, 2019). Internalization of these appearance ideals has been associated with eating pathology and extreme weight-control or exercise behaviours (Jankauskiene & Baceviciene, 2022). Furthermore, objectification and social appearance anxiety have been identified as important psychological factors associated with PSMU (Boursier et al., 2020), and it has been established that social media addiction is closely linked to appearance-focused social media consciousness (Çimke & Yıldırım Gürkan, 2023). Consistent with the I-PACE model, weight-related self-stigma and distress have been associated with emotional and cognitive responses that foster PSMU (Gan et al., 2025), while social withdrawal, emotional suppression, and negative body image may reinforce reciprocal associations between appearance concerns and problematic social media use (Li et al., 2024).

Although previous research has predominantly focused on bullying victimization, the present study specifically examines adolescents’ self-reported engagement in bullying perpetration. Evidence suggests that adolescents involved in bullying, particularly those who are experiencing victimization, frequently report greater loneliness and social isolation (Avcı & Yıldırım, 2014). Bullying involvement has been associated with greater loneliness (Eid et al., 2023), whereas parental and classmate support have been shown to buffer the ongoing course of bullying victimization over time (Healy et al., 2024).

Bullying related to physical appearance has also been associated with negative body image during adolescence. Teasing related to weight (Eisenberg et al., 2003; Haines et al., 2006; Puhl et al., 2013), facial features, and skin condition (Magin et al., 2008), as well as exclusion based on appearance, are widespread, and appearance-based bullying is particularly harmful for this age group. Repeated victimization may reinforce negative body image in some adolescent subgroups (Lee & Vaillancourt, 2019). Taken together, these findings—drawn largely from the bullying victimization literature—suggest that PSMU, bullying involvement broadly defined, and negative body image are closely interrelated and may jointly contribute to adolescents’ psychosocial difficulties. Thus, PSMU and bullying operate as intertwined risk factors that jointly threaten healthy adolescent development. The present study examines this link specifically through the lens of self-reported bullying perpetration rather than victimization.

### 1.1. The Mediating Role of Secure Attachment and Resilience

Within this multifaceted risk context, adolescents’ experiences of loneliness and negative body image may be associated with PSMU and bullying perpetration, while individual resources such as secure attachment and resilience may play an important role in these associations. Prior research has indicated that secure attachment is associated with lower levels of PSMU, with secure attachment orientation being negatively associated with social networking site addiction (Monacis et al., 2017). It is important to note, however, that in the present study, secure attachment was operationalized using a measure of generalized perceptions of security and trust in close relationships, rather than attachment specifically to parents or caregivers. Accordingly, references to “secure attachment” throughout this manuscript refer to this generalized interpersonal construct rather than parent–child or caregiver-specific attachment.

Accumulating evidence suggests that secure attachment is associated with lower levels of PSMU; for example, a secure attachment orientation is negatively associated with PSMU, with securely attached individuals appearing to be at lower risk of developing addictive social media use patterns (Monacis et al., 2017). In contrast, adolescents with insecure attachment styles tend to rely on maladaptive coping strategies, such as problem avoidance, which has been associated with a greater likelihood of problematic Internet-related behavioural addictions (Estevez et al., 2019). Although secure attachment is generally regarded as a relatively stable aspect of interpersonal functioning, adolescence is also a developmental period in which attachment-related perceptions and interpersonal experiences may remain open to change. Accordingly, in the present study, adolescents’ current perceptions of attachment security are considered an individual psychological resource that may statistically account for the associations between PSMU and psychosocial outcomes, without implying that PSMU causes changes in attachment security or that attachment security temporally precedes PSMU.

Although resilience has frequently been conceptualized as a moderator of the adverse effects of stress, previous studies have also supported its role as a mediator linking maladaptive behaviours with psychosocial outcomes. Accordingly, the present study conceptualized both resilience and secure attachment as statistical mediators to examine whether they account for the associations between PSMU and loneliness, bullying perpetration, and negative body image. Given the cross-sectional design, these indirect pathways should be interpreted as statistical associations rather than evidence of causal processes.

Overall, previous research suggests that secure attachment and resilience are important psychological resources associated with adolescents’ psychosocial adjustment. Examining their indirect roles may contribute to a better understanding of how PSMU is associated with loneliness, bullying perpetration, and negative body image.

### 1.2. The Present Study

Adolescents with higher levels of PSMU are associated with negative body image (Çimke & Yıldırım Gürkan, 2023), which may subsequently undermine their broader psychological well-being. However, individual psychological resources such as secure attachment patterns and resilience may help explain these statistical associations. To date, there has been a paucity of studies that simultaneously examine the indirect associations of secure attachment and resilience in the relationships between PSMU and loneliness, bullying perpetration, and negative body image. This gap limits the development of theoretically grounded and empirically informed psychosocial support programs for adolescents.

The present study tested a parallel mediation model to investigate whether secure attachment and resilience are statistically associated with the relationships between PSMU and three psychosocial outcomes: loneliness, bullying perpetration, and negative body image. Given the cross-sectional design, the proposed model was intended to examine indirect statistical associations rather than causal pathways.

The study is structured around three main hypotheses:

**H1.**  *PSMU is positively associated with loneliness, bullying perpetration, and negative body image.*

**H2.**  *Secure attachment and resilience are negatively associated with loneliness, bullying perpetration, and negative body image.*

**H3.**  *Secure attachment and resilience statistically mediate the associations between PSMU and loneliness, bullying perpetration, and negative body image.*

## 2. Method

### 2.1. Participants and Procedure

Participants were 712 high school students recruited from public high schools in a city in southeastern Türkiye, aged between 13 and 18 years (M_age_ = 15.33, SD = 1.13). The sample comprised 435 girls (61.1%) and 277 boys (38.9%). A cross-sectional study design was employed, and participants were recruited using a convenience sampling strategy. Participants were recruited through collaborating school administrations following approval from the Provincial Directorate of National Education. Regarding grade level, 27% of participants were in 9th grade, 31% in 10th grade, 23.3% in 11th grade, and 18.7% in 12th grade. More than half of the adolescents (50.8%) reported using social media for 2–3 h per day.

Before data collection, ethical approval was obtained from the Ethics Committee of Batman University (Ethics No: 2025/10-01), and institutional permission was granted by the Provincial Directorate of National Education (Approval No: 2025.030462.01). Parental consent forms were distributed through school administrations, and written informed consent was obtained from parents before participation. In addition, written assent was obtained directly from participants aged 13 to 17, and participants aged 18 provided their own written informed consent. Participants were informed of the study’s purpose, assured of data confidentiality, and notified that participation was voluntary and that no financial or other compensation was provided.

### 2.2. Measures

*The Bergen Social Media Addiction Scale (BSMAS):* The scale developed by Andreassen et al. (2016) and adapted to Turkish by Demirci (2019) was used to assess problematic social media use. The scale is unidimensional and consists of 6 items rated on a 5-point Likert scale (1 = very rarely, 2 = rarely, 3 = sometimes, 4 = often, 5 = very often). Total scores range from 6 to 30, with higher scores indicating higher levels of PSMU. An example item is, “Have you felt an increasing desire to use social media more and more?” There are no reverse-coded items. In the present study, the internal consistency coefficient (Cronbach’s alpha) was 0.79.

*Attachment Styles Scale: Short Form (ASQ-SF):* Secure attachment was assessed using the Short Form of the Attachment Styles Scale, originally developed by Feeney et al. (1994), shortened by Iwanaga et al. (2018), and adapted to Turkish by Çelik and Türk (2024). The short form comprises subscales for secure, anxious, and avoidant attachment. For this study, only the secure attachment subscale was used. Items are rated on a 6-point Likert scale (1 = strongly disagree, 2 = mostly disagree, 3 = somewhat disagree, 4 = somewhat agree, 5 = mostly agree, 6 = strongly agree). An example item is, “I am confident that other people will love and respect me.” There are no reverse-coded items; higher scores reflect higher levels of secure attachment. In the present sample, Cronbach’s alpha for the secure attachment subscale was 0.75. It should be noted that the ASQ-SF assesses generalized perceptions of security and trust in close relationships with other people in general, rather than attachment specifically to parents or caregivers; the items do not refer to caregivers, and no parent-specific attachment measure was administered in this study.

*Psychological Resilience Scale (PRS):* Psychological resilience was measured using the Psychological Resilience Scale developed by Merino and Privado (2015) and adapted to Turkish by Green et al. (2024). This brief instrument can be used with both adolescents and adults. The scale consists of 3 items rated on a 5-point Likert scale (1 = strongly disagree, 2 = disagree, 3 = neutral, 4 = agree, 5 = strongly agree). An example item is, “I become stronger when I face difficulties.” Higher scores indicate greater resilience; no items are reverse-coded. In the present study, Cronbach’s alpha was 0.83.

*Peer Bullying Scale (PBS):* Bullying perpetration was assessed with the Bullying Scale, developed by Shaw et al. (2013) to measure bullying behaviours among high school students and adapted to Turkish by Arslan (2017). The scale includes 10 items and has been analysed under a unidimensional structure. Responses are given on a 5-point Likert scale (1 = I did not do it, 2 = I did it once or twice, 3 = once a month, 4 = once a week, 5 = several times a week). Higher scores indicate more frequent engagement in bullying perpetration behaviours. An example item is, “I made fun of someone in a mean way.” In the present study, Cronbach’s alpha was 0.81. All items describe actions the respondent directed at others; the scale does not assess bullying victimization.

*Short Form of the UCLA Loneliness Scale (ULS-8):* Loneliness was measured using the Short Form of the UCLA Loneliness Scale (ULS-8), originally developed by Russell et al. (1978) and adapted into Turkish by Doğan et al. (2011). The scale consists of 8 items rated on a 4-point Likert scale (1 = not at all appropriate, 2 = somewhat inappropriate, 3 = appropriate, 4 = completely appropriate). Higher scores reflect higher levels of loneliness. An example item is, “There is no one I can turn to.” In the present study, Cronbach’s alpha was 0.76.

*Body Image Scale (BIS):* The Body Image Scale was developed by Saylan and Soyyiğit (2022) to assess body image among adolescents and young adults. The scale consists of 21 items with a 5-point Likert scale (1 = strongly disagree, 2 = disagree, 3 = neutral, 4 = agree, 5 = strongly agree) and includes four subscales: Negative Body Perception, Evaluation Sensitivity, Positive Body Perception, and Body Modification. In the present study, only the Negative Body Perception subscale was used. An example item is, “I feel disappointed when I look at myself in the mirror.” Higher scores indicate a more negative perception of the body. In the present study, Cronbach’s alpha was 0.88.

### 2.3. Data Analysis

First, descriptive statistics were computed, and the distributional characteristics of the data were evaluated by examining skewness and kurtosis coefficients to assess the normality assumption. Pearson correlation analyses were then performed to examine the interrelationships among the study variables.

Finally, parallel mediation analyses were conducted using PROCESS Macro 4 (Hayes, 2022) in IBM SPSS Statistics 24 to examine the indirect associations for secure attachment and resilience in the relationships between PSMU and loneliness, bullying perpetration, and negative body image. Indirect associations were evaluated using 5.000 bootstrap samples with 95% bias-corrected confidence intervals.

### 2.4. Covariates

In the present study, age, gender, grade level, and daily social media use duration were included as covariates in all analyses due to their potential associations with the dependent variables, namely bullying perpetration, loneliness, and negative body image (Field, 2024).

## 3. Results

### 3.1. Preliminary Analyses

Skewness coefficients ranged between −0.76 and 1.05, whereas kurtosis coefficients varied from −0.68 to 0.69 across all study variables, suggesting that the data satisfied acceptable normality criteria (|skewness|, |kurtosis| ≤ 2) (Tabachnick & Fidell, 2013). Confirmatory factor analyses (CFAs) supported the construct validity of the measurement instruments, with all scales demonstrating acceptable model fit. Following these preliminary analyses, Pearson correlation and parallel mediation analyses were conducted.

Pearson correlation analyses demonstrated that problematic social media use (PSMU) was significantly and negatively associated with secure attachment (*r* = −0.15, *p* < 0.001) and resilience (*r* = −0.19, *p* < 0.001), while showing significant positive associations with bullying perpetration (*r* = 0.38, *p* < 0.001), loneliness (*r* = 0.22, *p* < 0.001), and negative body image (*r* = 0.19, *p* < 0.001). In addition, secure attachment was inversely related to bullying perpetration (*r* = −0.24, *p* < 0.001), loneliness (*r* = −0.57, *p* < 0.001), and negative body image (*r* = −0.36, *p* < 0.001). Likewise, resilience demonstrated significant negative correlations with bullying perpetration (*r* = −0.25, *p* < 0.001), loneliness (*r* = −0.38, *p* < 0.001), and negative body image (*r* = −0.28, *p* < 0.001).

The internal consistency coefficients of the study measures ranged from 0.75 to 0.88, indicating satisfactory to strong reliability. Means, standard deviations, reliability coefficients, and Pearson correlation coefficients among the study variables are presented in Table 1.

### 3.2. Mediation Analyses

**Figure 1:**
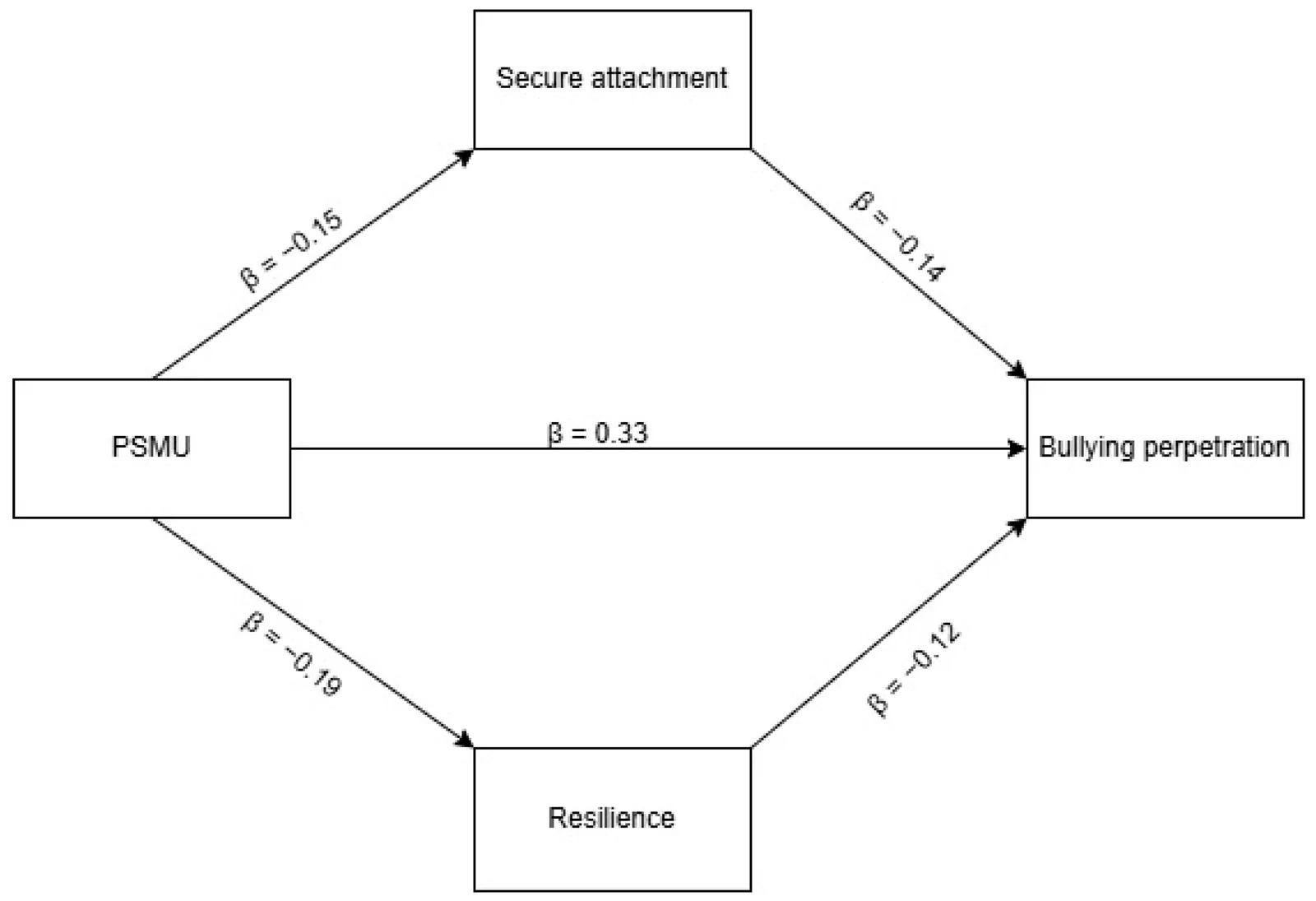
Parallel mediation model: Bullying perpetration. Note: Standardized path coefficients (β) are presented. All coefficients in the figure are statistically significant at *p* < 0.001.

**Figure 2:**
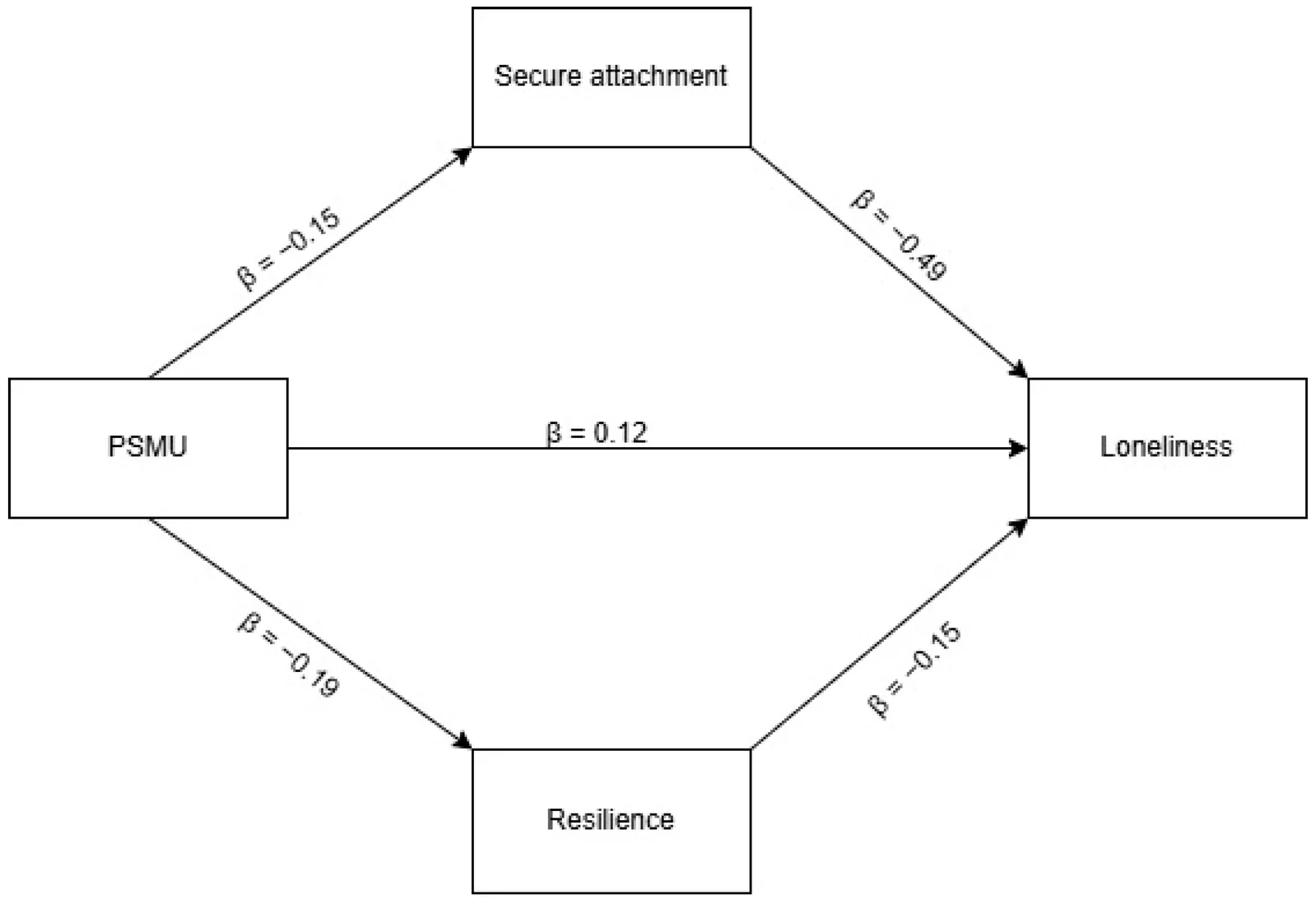
Parallel mediation model: Loneliness. Note: Standardized path coefficients (β) are presented. All coefficients in the figure are statistically significant at *p* < 0.001.

**Figure 3:**
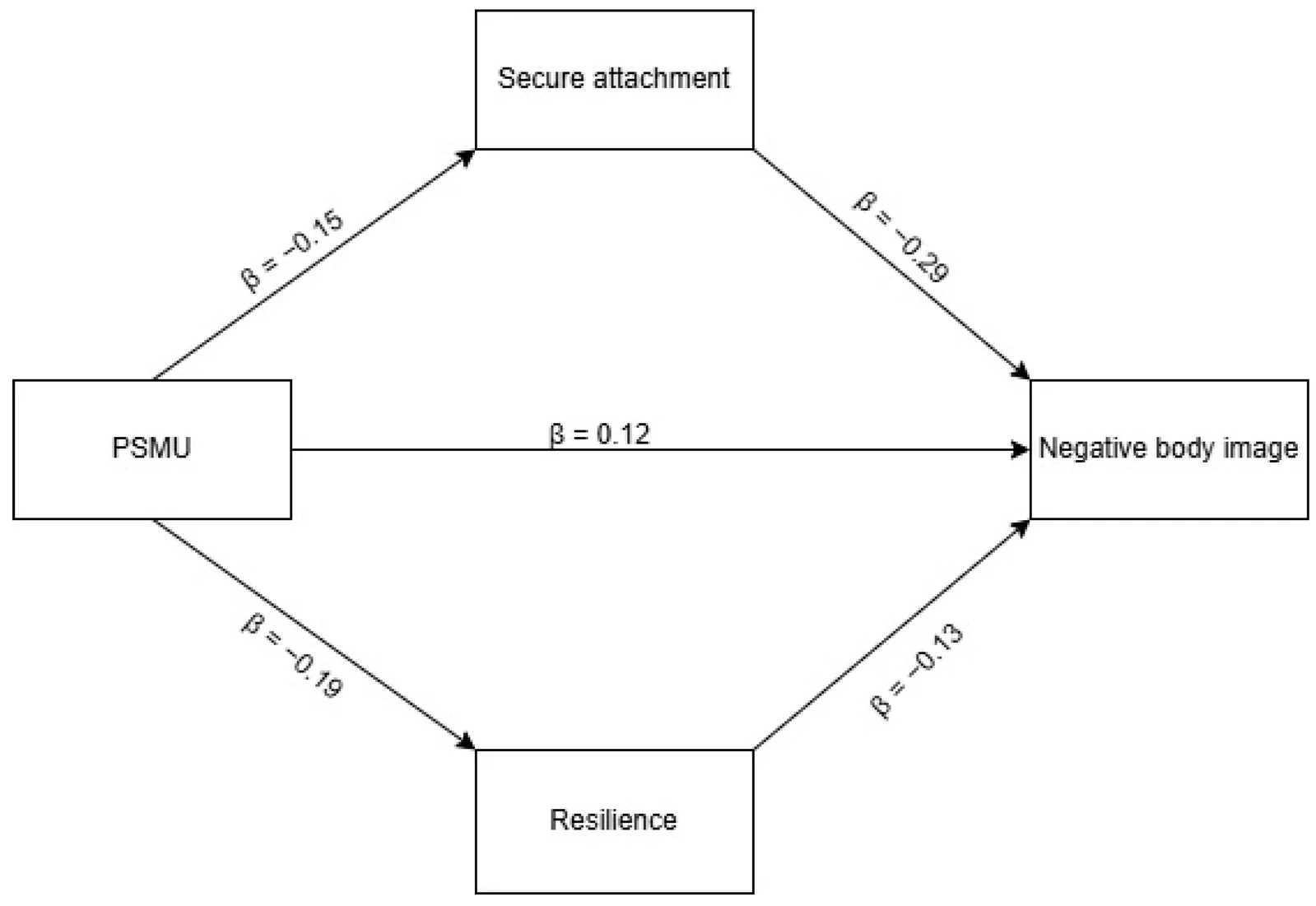
Parallel mediation model: Negative body image. Note: Standardized path coefficients (β) are presented. All coefficients in the figure are statistically significant at *p* < 0.001.

Following the preliminary analyses, parallel mediation analyses were conducted using PROCESS Macro 4 to examine the indirect associations between PSMU and bullying perpetration, loneliness, and negative body image through secure attachment and resilience. Standardized path coefficients are presented in Figure 1, Figure 2 and Figure 3, and detailed estimates are reported in Table 2 and Table 3.

Bootstrapping analyses (5000 resamples; 95% bias-corrected confidence intervals) indicated significant indirect associations between PSMU and bullying perpetration, loneliness, and negative body image through secure attachment and resilience. For bullying perpetration, the indirect associations through the mediators (secure attachment and resilience) were significant (effects = 0.022 and 0.020; 95% CIs [0.005, 0.005] and [0.043, 0.041], respectively). For loneliness, the indirect associations were likewise significant (effects = 0.079 and 0.025; 95% CIs [0.032, 0.009] and [0.129, 0.046], respectively). For negative body image, significant indirect associations were also observed (effect = 0.048 and 0.022; 95% CIs [0.019, 0.007] and [0.082, 0.043], respectively). The total indirect associations of PSMU were significant for bullying perpetration (effect = 0.042, 95% CI [0.017, 0.073]), loneliness (effect = 0.104, 95% CI [0.048, 0.165]), and negative body image (effect = 0.071, 95% CI [0.032, 0.112]) (see Table 3).

Collectively, the findings suggest that secure attachment and resilience statistically accounted for part of the associations between PSMU and bullying perpetration, loneliness, and negative body image. Given the cross-sectional design, these indirect associations should not be interpreted as evidence of causal pathways.

## 4. Discussion

The present study examined the indirect roles of secure attachment and resilience in the relationships between PSMU and loneliness, bullying perpetration, and negative body image among adolescents. The findings were generally consistent with the proposed hypotheses. They supported the hypothesized patterns of statistical associations among the study variables. Specifically, higher levels of PSMU were positively associated with loneliness, bullying perpetration, and negative body image (H1), whereas secure attachment and resilience were negatively related to these outcomes (H2). Furthermore, secure attachment and resilience showed significant indirect associations in the relationships between PSMU and loneliness, bullying perpetration, and negative body image (H3).

Adolescence is a developmental period in which identity formation, the pursuit of social approval, and peer relationships are particularly salient (Li et al., 2024; Lu et al., 2025; Sala et al., 2024). In this sensitive context, excessive and dysfunctional social media use may exacerbate psychosocial vulnerabilities. Consistent with our first hypothesis, PSMU was positively and significantly associated with loneliness, bullying perpetration, and negative body image. Specifically, adolescents reporting higher levels of PSMU also reported greater loneliness, greater engagement in bullying perpetration, and more negative evaluations of their bodies. This pattern is consistent with previous research documenting positive associations between PSMU and loneliness (Wu et al., 2024; Yam et al., 2024), bullying perpetration (Butasım & Erdoğdu, 2025), and negative body image (Chua & Chang, 2016; Çimke & Yıldırım Gürkan, 2023; Gan et al., 2025).

For instance, adolescents who engage in excessive and dysfunctional social media use tend to report higher levels of loneliness (Błachnio et al., 2016), display greater tendencies toward bullying behaviours (Craig et al., 2020; Parris et al., 2022), and experience increased body dissatisfaction due to upward social comparisons in online contexts (Çimke & Yıldırım Gürkan, 2023; Gan et al., 2025; Saiphoo & Vahedi, 2019). At the same time, previous studies also indicate that bullying perpetration (Butasım & Erdoğdu, 2025), loneliness (Wu et al., 2024; Yam et al., 2024), and negative body image (Li et al., 2024) are also associated with higher levels of PSMU, suggesting potentially reciprocal associations. This bidirectionality can be understood in light of the compensatory internet use model (Kardefelt-Winther, 2014), which posits that individuals turn to the online environment to escape from real-life problems they cannot cope with, inadvertently exacerbating those problems. Accordingly, the present findings, together with previous research, suggest that the associations between PSMU, loneliness, bullying perpetration, and negative body image may be reciprocal.

Securely attached adolescents tend to experience more harmonious relationships with family and peers, hold more positive views of themselves and others, and report fewer social problems (Kesebir et al., 2011). Our findings are consistent with this perspective and support the second hypothesis, indicating that secure attachment was negatively associated with loneliness, bullying perpetration, and negative body image. Specifically, adolescents reporting higher levels of secure attachment also reported lower levels of loneliness, engagement in bullying perpetration, and negative body image. These findings are consistent with previous research suggesting that secure attachment is associated with lower levels of body image difficulties (Szalai et al., 2017), bullying (Kokkinos, 2013), and loneliness (DiTommaso et al., 2003).

Parental attachment quality and self-concept clarity are reciprocally related during adolescence (Wang et al., 2024). Adolescents with secure attachment are more likely to build supportive relationships and resist negative peer influences (Allen et al., 2007). Consequently, securely attached adolescents tend to exhibit higher social competence and lower loneliness (DiTommaso et al., 2003) and engage less frequently in bullying perpetration behaviours (Yüksel Doğan & Demircioğlu, 2025).

Consistent with previous work, resilience in the present study was negatively associated with loneliness (Yam et al., 2024) and bullying perpetration (Simsek et al., 2025), as well as negative body image (Borinsky et al., 2019). Resilience is widely recognised as an important psychological resource that supports adaptation to stressful life experiences (Masten, 2001). Taken together, these findings suggest that both secure attachment and resilience may represent important psychological resources associated with lower levels of loneliness, bullying perpetration, and negative body image among adolescents.

Finally, the mediation analyses revealed significant indirect statistical associations between PSMU, loneliness, bullying perpetration, and negative body image, with lower levels of secure attachment and resilience emerging as statistical correlates within these associations (H3). These findings suggest that secure attachment and resilience may partially account for the associations between PSMU and adolescents’ psychosocial functioning. Adolescents reporting lower levels of secure attachment also reported more psychosocial difficulties associated with PSMU (Musetti et al., 2022), whereas those with higher levels of secure attachment reported fewer such difficulties. Previous research similarly indicates that secure attachment and resilience are inverse correlates of negative body image (Szalai et al., 2017), bullying (Kokkinos, 2013), and loneliness (DiTommaso et al., 2003).

It should also be noted that PSMU explained only a small proportion of the variance in secure attachment (2%) and resilience (3%), suggesting that these psychological resources are influenced by numerous developmental, familial, and contextual factors beyond problematic social media use. Accordingly, although the observed indirect associations were statistically significant, their practical significance should be interpreted with caution.

### 4.1. Implications

The association between problematic Internet use and resilience (Hidalgo-Fuentes et al., 2023), together with the inverse associations of resilience with adverse mental health outcomes (Luo et al., 2025; Ciren et al., 2025) and of secure attachment with negative emotional outcomes in the context of adverse experiences (Darling Rasmussen et al., 2019), constitutes one of the more extensively investigated areas within the existing literature. The present study extends this rich field by demonstrating significant indirect associations of resilience and secure attachment in the relationships between PSMU and loneliness, bullying perpetration, and negative body image among adolescents.

By showing that both secure attachment and resilience were indirectly associated with these relationships, our findings suggest that secure attachment and resilience may represent psychological resources statistically associated with lower levels of these psychosocial difficulties linked to PSMU, although this interpretation remains tentative given the cross-sectional design. From an applied standpoint, a better understanding of the nature of these associations may help inform, rather than directly establish, preventive strategies targeting loneliness, bullying perpetration, and negative body image, pending confirmation through longitudinal or experimental research.

The results indicate that higher levels of PSMU were associated with greater loneliness, engagement in bullying perpetration, and negative body image. Although these cross-sectional findings do not establish causal relationships, they highlight secure attachment and resilience as psychological resources associated with these psychosocial outcomes.

In practice, programs designed to strengthen adolescents’ resilience and promote secure attachment patterns may represent promising components of prevention efforts addressing the psychosocial difficulties associated with PSMU. However, because the present cross-sectional design cannot demonstrate that increasing attachment security or resilience would actually reduce PSMU-related difficulties, such recommendations remain speculative, and their effectiveness would need to be established through dedicated intervention research. Because the present measure assessed generalized perceptions of interpersonal security rather than attachment specifically to parents or caregivers, we refrain from recommending parent-child attachment interventions on the basis of these findings; such recommendations would require validation using measures specifically designed to assess caregiver attachment. Potentially relevant approaches may instead include school-based interventions to enhance emotion regulation, coping skills, and broader social support, which could help adolescents build a stronger general sense of trust and security in their relationships with others. Training and awareness programs for teachers, school counsellors, and policymakers could similarly highlight the potential relevance of resilience and attachment to the psychosocial risks associated with PSMU, pending further empirical support.

### 4.2. Limitations and Future Directions

The present study has several limitations. First, the sample consisted exclusively of adolescents, which may limit the generalizability of the results to individuals from different developmental periods, educational backgrounds, or sociocultural contexts. Future research employing more diverse samples across varying age groups and cultural settings would contribute to enhancing the external validity and broader applicability of the findings.

Second, the cross-sectional nature of the study design limits the ability to draw causal conclusions, as the directionality and temporal ordering of the observed associations cannot be established with clarity, and the possibility of reverse causality cannot be ruled out—for example, higher levels of loneliness, bullying perpetration, and negative body image may also be associated with increased PSMU. Future longitudinal research is required to establish the temporal sequence and direction of the relationships among these variables.

Third, the study relied exclusively on self-report instruments, which may be vulnerable to various sources of response bias, including social desirability tendencies, inaccurate self-perceptions, misinterpretation of items, and potential over- or under-reporting of experiences. Future research may benefit from incorporating alternative or multi-method data collection approaches, such as observational methods, peer or parent reports, and longitudinal assessments, to strengthen the robustness and validity of the findings.

Fourth, a further limitation concerns the bullying measure itself. The instrument used in the present study assessed only self-reported bullying perpetration (e.g., making fun of someone), not bullying victimization; consequently, the findings speak exclusively to adolescents’ own engagement in bullying behaviours and cannot be generalized to experiences of being bullied. Given that much of the broader literature on bullying and adjustment concerns victimization rather than perpetration, future research incorporating both constructs would help clarify whether the present pattern of associations extends to victimization as well.

Fifth, although the mediation analyses adjusted for age, gender, grade level, and daily social media use, other potentially relevant demographic and contextual covariates—such as socioeconomic status and family characteristics—were not assessed in the present study. Future research should examine whether these associations remain robust after controlling for these additional covariates.

Finally, although PROCESS Macro is a well-established approach for testing mediation models with observed variables, future research could employ structural equation modelling (SEM) to simultaneously estimate all hypothesized pathways while accounting for measurement error and the correlations among the outcome variables.

## 5. Conclusions

This study provides empirical evidence regarding the associations among PSMU, secure attachment, resilience, loneliness, bullying perpetration, and negative body image during adolescence. The findings indicate that PSMU was positively associated with loneliness, bullying perpetration, and negative body image, whereas secure attachment and resilience were negatively associated with these psychosocial difficulties. Furthermore, the findings suggest that secure attachment and resilience may partially account for the statistical associations between PSMU and adolescents’ psychosocial adjustment.

For mental health practitioners and educational professionals, these findings highlight the potential value of interventions that address PSMU and its associated psychosocial difficulties while also promoting secure attachment and strengthening resilience. Programs that promote adaptive coping and emotion regulation and enhance social connectedness may be especially valuable. Because the present attachment measure assessed generalized interpersonal security rather than attachment specifically to parents or caregivers, we do not extend this recommendation to family- or caregiver-focused attachment interventions without further validation using measures designed specifically for that purpose. Nevertheless, because of the cross-sectional design, the present findings should be interpreted as statistical associations rather than evidence of causal relationships. Future longitudinal and intervention studies are needed to clarify the temporal ordering and causal mechanisms underlying these associations.

## Figures and Tables

**Table 1:** Descriptive statistics and correlation matrix.

	Descriptive Statistics					Correlations					
Variable	Mean	SD	Skewness	Kurtosis	α	1.	2.	3.	4.	5.	6.
1. PSMU	17.42	6.00	−0.01	−0.68	0.79	—	−0.15 **	−0.19 **	0.38 **	0.22 **	0.19 **
2. Secure attachment	16.65	4.83	−0.45	−0.09	0.75	—	—	0.43 **	−0.24 **	−0.57 **	−0.36 **
3. Resilience	11.14	3.29	−0.76	−0.09	0.83	—	—	—	−0.25 **	−0.38 **	−0.28 **
4. Bullying perpetration	15.12	5.79	1.03	0.69	0.81	—	—	—	—	0.29 **	0.17 **
5. Loneliness	13.85	4.97	1.05	0.65	0.76	—	—	—	—	—	0.43 **
6. Negative body image	14.92	6.75	0.79	0.02	0.88	—	—	—	—	—	—

** All correlation coefficients are significant at the *p* < 0.001 level.

**Table 2:** Unstandardized coefficients for the proposed mediation model.

	Consequent			
	*M*_1_ (Secure attachment)			
Antecedent	Coeff.	*SE*	*t*	*p*
*X* (PSMU)	−0.121	0.030	−4.040	<0.001
Constant	18.770	0.555	33.780	<0.001
	*R*^2^ = 0.02 *F* = 23.35; *p* < 0.001			
	*M*_2_ (Resilience)			
Antecedent	Coeff.	*SE*	*t*	*p*
*X* (PSMU)	−0.107	0.020	−5.262	<0.001
Constant	12.976	0.377	34.347	<0.001
	*R*^2^ = 0.03 *F* = 27.69; *p* < 0.001			
	*Y*_1_ (Bullying perpetration)			
Antecedent	Coeff.	*SE*	*t*	*p*
*X* (PSMU)	0.320	0.033	9.705	<0.001
*M*_1_ (Secure attachment)	−0.164	0.044	−3.708	<0.001
*M*_2_ (*Resilience*)	−0.220	0.065	−3.382	<0.001
Constant	14.666	1.085	13.512	<0.001
	*R*^2^ = 0.19 *F* = 56.69; *p* < 0.001			
	*Y*_2_ (Loneliness)			
Antecedent	Coeff.	*SE*	*t*	*p*
*X* (PSMU)	0.100	0.025	3.937	<0.001
*M*_1_ (Secure attachment)	−0.504	0.0341	−14.812	<0.001
*M*_2_ (*Resilience*)	−0.227	0.050	−4.534	<0.001
Constant	12.10	0.96	12.53	<0.001
	*R*^2^ = 0.36 *F* = 137.73; *p* < 0.001			
	*Y*_3_ (Negative body image)			
Antecedent	Coeff.	*SE*	*t.*	*p*
*X* (PSMU)	0.139	0.039	3.543	<0.001
*M*_1_ (Secure attachment)	−0.406	0.052	−7.708	<0.001
*M*_2_ (*Resilience*)	−0.265	0.077	−3.420	<0.001
Constant	22.200	1.291	17.195	<0.001
	*R*^2^ = 0.17 *F* = 48.10; *p* < 0.001			

Note: Number of bootstrap samples for percentile bootstrap confidence intervals: 5000. *SE* = standard error; Coeff = unstandardized coefficient; *X* = independent variable; *M* = mediator variable; *Y* = outcome variable.

**Table 3:** Standardized total and indirect effects of the tested research model (number of bootstrap samples: 5000).

Path	Effect	*SE*	BootLLCI	BootULCI
Total effect PSMU → Bullying perpetration	0.042	0.014	0.017	0.073
Indirect effect				
PSMU → Secure attachment → Bullying perpetration	0.022	0.009	0.005	0.043
PSMU → Resilience → Bullying perpetration	0.020	0.009	0.005	0.041
Total effect				
PSMU → Loneliness	0.104	0.029	0.048	0.165
Indirect effect				
PSMU → Secure attachment → Loneliness	0.079	0.024	0.032	0.129
PSMU → Resilience → Loneliness	0.025	0.009	0.009	0.046
Total effect				
PSMU → Negative body image	0.071	0.020	0.032	0.112
Indirect effect				
PSMU → Secure attachment → Negative body image	0.048	0.016	0.019	0.082
PSMU → Resilience → Negative body image	0.022	0.009	0.007	0.043

## Data Availability

The datasets generated and/or analysed during the current study are available from the corresponding author upon reasonable request.
